# Determinants and temporal trajectories of exclusive breastfeeding cessation in 16 sub-Saharan African countries: a pooled multivariable survival analysis of the demographic and health survey (2019–2024)

**DOI:** 10.1186/s12889-026-28431-9

**Published:** 2026-07-15

**Authors:** Samson Shumba, Douglas Nkumbwa, Violet Kayamba, Mwila Kapembwa

**Affiliations:** 1 Department of Epidemiology and Biostatistics, School of Public Health, University of Zambia, Lusaka, Zambia; 2 Copper Rose Zambia, Lusaka, Zambia; 3 Department of Economics, School of Humanities and Social Sciences, University of Zambia, Lusaka, Zambia; 4 Tropical Gastroenterology and Nutrition Group, School of Medicine, University of Zambia, Lusaka, Zambia; 5 Institute of Biomedical and Health Sciences, Mukuba University, Kitwe, Zambia

**Keywords:** Exclusive breastfeeding, Breastfeeding cessation, Time-to-event analysis, Cox proportional hazards model, Gompertz parametric model, Temporal trajectory, Sub-Saharan Africa

## Abstract

**Background:**

Exclusive breastfeeding (EBF) for the first six months of life is the globally recommended standard of infant nutrition, yet early cessation remains prevalent and highly variable across sub-Saharan Africa (SSA). Despite growing evidence on its determinants, few studies have applied pooled survival analysis with community-level contextual variables to examine both the timing and determinants of EBF cessation across multiple countries simultaneously. This study aimed to determine the time to EBF cessation and its associated factors among infants across 16 SSA countries, and to characterize the country-specific temporal trajectories of cessation hazard.

**Methods:**

We analysed pooled Demographic and Health Survey (DHS) data from 16 SSA countries (2019–2024). The analytical sample comprised 20,830 last-born infants (weighted *N* = 20,085) aged 0–5 months. Cessation of EBF was the time-to-event outcome, defined via 24-hour dietary recall. Kaplan–Meier curves and log-rank tests were used for descriptive survival analysis. A multivariable Cox proportional hazards model stratified by country and adjusted for primary sampling unit clustering was fitted to identify independent determinants of EBF cessation. A Gompertz parametric model was additionally fitted, yielding country-specific shape parameters (γ) that characterise the temporal trajectory of cessation hazard within each country.

**Results:**

Overall, 75.74% of infants were exclusively breastfed for up to five months, with a pooled cessation incidence rate of 0.093 per person-month (95% CI: 0.090–0.095) across 51,047 person-months of observation. Country-specific incidence rates ranged from 0.020 per person-month in Rwanda to 0.195 per person-month in Kenya, reflecting substantial cross-country heterogeneity. Kaplan–Meier analysis revealed that the probability of remaining exclusively breastfed declined from 0.971 at month 1 to 0.360 at month 5, with the steepest decline occurring between months three and five. In the adjusted Cox model, five independent predictors were identified: higher maternal education (aHR = 0.82; 95% CI: 0.69–0.98), Muslim religious affiliation (aHR = 0.73; 95% CI: 0.60–0.88), and early breastfeeding initiation within one hour of birth (aHR = 0.89; 95% CI: 0.84–0.94) were independently protective against cessation, while high birth weight (≥ 4,000 g) (aHR = 1.22; 95% CI: 1.07–1.39) and attendance at eight or more antenatal care visits (aHR = 1.16; 95% CI: 1.03–1.30) were associated with greater cessation hazard. No community-level variable reached statistical significance in the adjusted model. Gompertz analysis revealed significantly increasing cessation hazard over time in 11 of 16 countries, with Rwanda recording the steepest temporal acceleration (γ = 1.299; *p* = 0.015); four countries showed no statistically detectable change in hazard across the observation period.

**Conclusions:**

EBF cessation in SSA is driven primarily by individual-level factors, with the period between three and five months representing a critical window of vulnerability in most countries. Beyond identifying determinants of cessation, this study demonstrates substantial cross-country variation in the temporal trajectory of cessation risk. The country-specific Gompertz shape parameters provide novel evidence on when the hazard of EBF cessation accelerates, offering temporal insights that conventional prevalence-based analyses cannot capture. These findings suggest that breastfeeding interventions should not only target high-risk populations but also be timed according to country-specific cessation trajectories. Strengthening early breastfeeding initiation, integrating breastfeeding counselling into antenatal and postnatal care, and providing targeted support for mothers of high-birth-weight infants may improve EBF retention across SSA.

**Supplementary Information:**

The online version contains supplementary material available at https://doi.org/10.1186/s12889-026-28431-9.

## Background

Breast milk is the optimal food for infants in the first months of life, providing the complete nutritional, immunological, and developmental substrate required for healthy growth [1]. Exclusive breastfeeding (EBF), defined as feeding infants only breast milk for the first six months of life, without introducing any additional food or liquid is the globally recommended standard of infant nutrition [2]. The evidence base for EBF is extensive: it substantially reduces infant mortality from infectious diseases, confers protection against pneumonia, diarrhoeal disease, and asthma, and supports cognitive development [2–4]. Beyond infancy, breastfed individuals demonstrate lower blood pressure, reduced cholesterol levels, and a decreased risk of type 2 diabetes mellitus in adulthood [5]. For mothers, breastfeeding reduces the risk of breast and ovarian cancers and supports birth spacing [6]. These benefits are not merely additive but synergistic, making EBF one of the most cost-effective preventive health interventions available to low- and middle-income countries [6].

Despite this evidence, global EBF rates remain far below recommended targets. The World Health Assembly (WHA) first set a target of 50% EBF prevalence among infants under six months by 2025; recognising slow global progress, WHO Member States at the 78th World Health Assembly in 2025 revised this target upward to 60% by 2030 as part of the updated Global Nutrition Targets framework [7]. The WHO currently estimates that only approximately 44% of infants aged 0–6 months are exclusively breastfed worldwide, with suboptimal practices associated with approximately 820,000 child deaths annually [3]. The EBF target is embedded within Sustainable Development Goal (SDG) 2.2, which calls for ending all forms of malnutrition by 2030, and is intrinsically linked to SDG 3 targets on child mortality reduction and noncommunicable disease prevention [8]. Given the well-documented socioeconomic and geographic disparities in breastfeeding outcomes, improving EBF rates also carries direct relevance for SDG 10 (Reduced Inequalities) [9, 10].

In sub-Saharan Africa (SSA), breastfeeding initiation is nearly universal, yet adherence to EBF over the first six months remains suboptimal and highly variable across countries. Recent analyses of Demographic and Health Survey (DHS) data estimate that only approximately 41%–49% of infants aged 0–5 months in SSA are exclusively breastfed, with WHO regional estimates placing the average at approximately 41.9% [11, 12]. Country-level disparities are substantial ranging from below 20% in some settings to above 80% in others reflecting the diverse cultural, social, and health system contexts that shape infant feeding practices across the region [13, 14]. These disparities are particularly consequential given that SSA bears a disproportionate burden of preventable child mortality, and suboptimal EBF represents a critical yet modifiable contributor to this burden [6].

The determinants of EBF cessation in SSA operate across multiple levels. At the individual level, maternal age, educational attainment, employment status, antenatal care (ANC) attendance, and timing of breastfeeding initiation have been consistently associated with EBF duration [15, 16]. Household- and community-level factors, including partner and family support, sociocultural norms around infant feeding, and access to health services, further shape breastfeeding trajectories [11, 17, 18]. Structural barriers such as inadequate maternity protection legislation, weak workplace breastfeeding policies, and commercial infant formula marketing compound these influences, particularly in urban settings [6]. Socioeconomic inequalities intersect with all of these pathways, generating marked within- and between-country variation in breastfeeding outcomes [9, 10].

Community-level characteristics are theorised to shape individual breastfeeding behaviour through several intersecting pathways. Community educational attainment may influence EBF through normative mechanisms: in communities where higher education is prevalent, breastfeeding norms, health literacy, and receptiveness to evidence-based infant feeding guidance may be collectively reinforced, creating a social environment conducive to EBF continuation [6]. Community wealth operates through structural pathways, wealthier communities tend to have better access to health facilities, more skilled birth attendants, and greater availability of postnatal lactation support services, all of which have been shown to independently predict EBF duration [2]. Community employment levels may create competing demands on mothers’ time through social norms around female labour participation, reducing the feasibility of sustained EBF even among women who are not themselves formally employed. Community media exposure may function as a dual-edged pathway: while mass media can disseminate health promotion messaging that supports EBF, it simultaneously increases exposure to commercial infant formula marketing, which is a well-documented driver of early cessation [6]. These pathways are inherently indirect and context-dependent, which may partly explain why community-level effects attenuate after individual-level adjustment, a finding consistent with the hierarchical nature of multilevel determinants of health behaviour.

Despite this growing evidence base, important methodological gaps remain. Most existing multi-country studies rely on cross-sectional prevalence estimates that obscure the temporal dynamics of cessation and cannot identify the specific periods within early infancy at which cessation risk peaks. Survival analysis methods which explicitly model time to event, account for right-censoring, and accommodate the clustered, stratified sampling structure of DHS data are better suited to this research question, yielding more precise and unbiased estimates of cessation risk and its predictors than cross-sectional approaches [15, 16]. Yet few studies have applied pooled survival analysis with community-level contextual variables to rigorously examine EBF cessation timing and its determinants across multiple SSA countries simultaneously [19].

This study addresses these gaps by analysing pooled DHS data from 16 SSA countries (2019–2024) using a multivariable Cox proportional hazards model stratified by country alongside a Gompertz parametric sensitivity analysis. The dual-model approach yields both common adjusted hazard ratios for predictors across the pooled sample and country-specific Gompertz shape parameters (γ) that characterise the temporal trajectory of cessation hazard within each country, providing aggregate and country-level programmatic intelligence on when, not just among whom, EBF cessation is most likely to occur. Findings are intended to inform targeted, evidence-based policy and programmatic responses aligned with the SDG 2.2, SDG 3, and SDG 10 frameworks.

## Methods

### Data source

This study used pooled data from the Demographic and Health Surveys (DHS) conducted between 2019 and 2024 across 16 sub-Saharan African countries: Angola, Burkina Faso, Côte d’Ivoire, the Democratic Republic of Congo, Gambia, Ghana, Kenya, Lesotho, Madagascar, Malawi, Mali, Mozambique, Nigeria, Rwanda, Tanzania, and Zambia (Table 1). Data were extracted from the Children’s Recode (KR) files, which contain information on births, infant feeding practices, and child health indicators collected from women aged 15–49 years. The DHS Programme conducts nationally representative household surveys using a two-stage stratified cluster sampling design, with enumeration areas (EAs) selected randomly at the first stage and households selected systematically within each EA at the second stage. All 16 surveys were conducted under the standard DHS Round 8 protocol, ensuring harmonized questionnaire content, data collection procedures, and variable coding across countries, which supports the validity of cross-country pooled analysis.

**Table 1:** DHS survey years for the 16 sub-Saharan African countries included in the pooled analysis

No.	Country	DHS Survey Year	*n* (weighted)
1	Angola	2023–2024	1262
2	Burkina Faso	2021	1254
3	Congo DR	2023–2024	2277
4	Côte d’Ivoire	2021	985
5	Gambia	2019–2020	897
6	Ghana	2022	828
7	Kenya	2022	1711
8	Lesotho	2023–2024	258
9	Madagascar	2021	1262
10	Malawi	2024	1179
11	Mali	2023–2024	1717
12	Mozambique	2022–2023	997
13	Nigeria	2024	2727
14	Rwanda	2019–2020	781
15	Tanzania	2022	1105
16	Zambia	2024	844
**Total**			**20,085**

*DHS *Demographic and Health Survey. Survey years sourced from the DHS Program official publications and the Global Health Data Exchange (GHDx)

### Participant selection

**Fig. 1:**
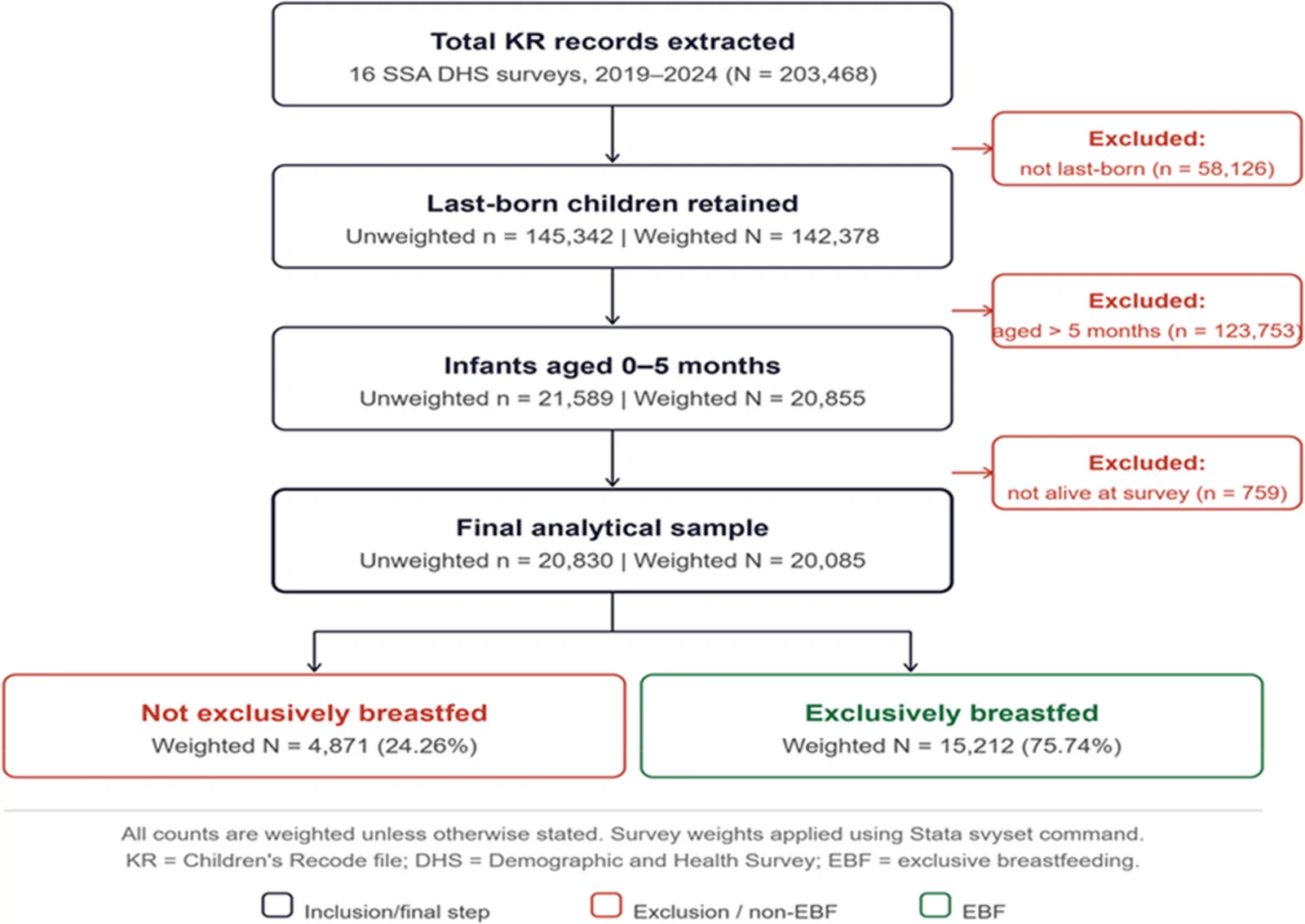
Participant selection flow diagram for the pooled survival analysis of exclusive breastfeeding cessation across 16 sub-Saharan African countries, DHS 2019–2024

Of 203,468 Children’s Recode records extracted from 16 DHS surveys, 58,126 non-last-born children, 123,753 infants aged more than five months, and 759 children who were not alive at the time of survey were sequentially excluded. The final analytical sample comprised 20,830 unweighted observations (weighted *N* = 20,085), of whom 15,212 (75.74%) were exclusively breastfed and 4,871 (24.26%) were not exclusively breastfed at the time of survey (see Fig. 1 below).

### Outcome variable

The outcome variable was the cessation of EBF among infants aged 0–5 months, which was assessed via 24-hour dietary recall data consistent with the DHS standard definitions. A time-to-event outcome was constructed using the child’s age in months (DHS variable b19) as the analysis time variable. Cessation of EBF was coded as 1 (failure) for infants who were not exclusively breastfed and 0 (right-censored) for those who remained exclusively breastfed at the time of the survey. The analytical sample was restricted to last-born children aged 0–5 months who were alive at the time of the survey, consistent with DHS analytical conventions. In the DHS Children’s Recode (KR) file, each record represents one child born to an interviewed woman, with one record retained per last-born child to avoid within-mother correlation; restricting to last-born infants therefore yields an analytically independent and age-eligible sample.

### Explanatory variables

#### Community-level factors

Community-level variables were derived by aggregating individual-level responses within the enumeration areas (EAs). Four community-level indicators were constructed: community wealth (proportion of poor households in the EA), community educational attainment (proportion of women with secondary or higher education), community employment (proportion of women in paid work), and community media exposure (proportion with mass media access). Each indicator was categorized into low, medium, and high tertiles using the *xtile* command in Stata, with tertile cut-points derived from the pooled distribution of cluster-level proportions across all 16 countries rather than within individual countries, ensuring a common classification threshold for cross-country comparability.

### Statistical analysis

All analyses were performed in Stata 14.2, with statistical significance set at α = 0.05 (two-sided). DHS survey weights were applied throughout using the *svyset* command, specifying the two-stage stratified design with primary sampling units (PSUs), strata, and probability weights. Weighted frequencies and percentages were computed for all categorical variables, and bivariate associations were assessed using design-adjusted Pearson chi-square tests. Kaplan–Meier survival curves were generated and group differences evaluated via log-rank tests. As the Kaplan–Meier survival curve was not expected to cross the 0.50 threshold given high overall EBF retention, the restricted mean survival time (RMST), defined as the area under the survival curve from month 0 to month 5 was computed using the *stci*,* rmean* command in Stata 14.2 and reported by country as a summary measure of average EBF duration. Person-time was calculated as each infant’s age in completed months at survey, with non-exclusively breastfed infants contributing person-time to their age at survey and exclusively breastfed infants right-censored at their current age. The incidence rate of EBF cessation was expressed per person-month.

The time-to-event outcome was defined using each infant’s age in completed months (DHS variable *b19*), following a current-status (cross-sectional) survival design in which the precise cessation time is unobserved. Under this approach, infants not exclusively breastfeeding at survey are treated as having ceased EBF at their current age (interval-censored within the preceding monthly interval), while those still exclusively breastfeeding are right-censored. Although this may introduce misclassification of event timing, it is methodologically consistent with established DHS-based breastfeeding survival analyses [11, 15].

A Cox proportional hazards model was fitted, stratified by country to allow country-specific baseline hazard functions while estimating pooled covariate-level hazard ratios. Country stratification was preferred over fixed- or random-effects specifications because the country variable violated the proportional hazards assumption (Schoenfeld test *p* < 0.05 prior to stratification), and because fitting shared frailty models across 16 complex survey designs posed substantial convergence challenges. The global Schoenfeld test for the final model was non-significant (*p* = 0.243).

Weibull, Gompertz, log-logistic, and log-normal parametric models were compared using AIC and BIC; the Gompertz model provided the best fit and was used as a sensitivity analysis. Unlike the primary Cox model, the Gompertz model included country as a fixed-effect indicator (Angola as reference), yielding country-specific shape parameters characterising the temporal trajectory of cessation hazard. Given low overall missingness (3.6%), complete-case analysis was considered unlikely to materially affect estimates.

Post-hoc power estimation was conducted using Stata’s stpower cox command based on Freedman’s method. With a total sample of 20,085 participants, an observed event fraction of 24.4% (yielding an estimated 4,901 cessation events), and a two-sided α = 0.05, the study achieves greater than 99% power to detect hazard ratios of 1.20 or greater for binary predictors with approximately equal exposure prevalence (SD = 0.50). The study achieves approximately 77% power to detect hazard ratios as small as 1.08. The study is therefore adequately powered to detect hazard ratios of modest to moderate magnitude, consistent with the precision of confidence intervals observed across the five significant predictors in the adjusted model. Multicollinearity was assessed using variance inflation factors (VIF < 5 threshold); no problematic multicollinearity was detected (mean VIF = 1.97). No formal correction for multiple comparisons was applied, as all 18 predictors were selected a priori on theoretical and empirical grounds prior to analysis rather than identified through data-driven exploration. However, with 18 predictors tested simultaneously at α = 0.05, the probability of at least one spurious significant association by chance alone is non-trivial, and findings from the five significant predictors should be interpreted with appropriate caution and replicated in independent samples before informing policy.

### Individual-level factors

Individual-level variables were selected on the basis of their theoretical relevance and prior empirical evidence. These included maternal age group, place of residence, educational attainment, marital status, employment status, household wealth index, parity, media exposure, type of birth, sex of the child, perceived size at birth, timing of breastfeeding initiation, number of ANC visits, and religious affiliation. The definitions and measurement categories for each variable are presented in Table 1.

### Bias

Several potential sources of bias were considered and addressed in the analytical design. To minimise interval censoring arising from the cross-sectional current-status survival design, event timing was approximated using each infant’s age in completed months at survey, consistent with established DHS-based breastfeeding survival analyses. Survey weights were applied throughout using the Stata *svyset* command to correct for unequal probabilities of selection arising from the complex two-stage stratified sampling design, and robust standard errors clustered at the primary sampling unit level were used to account for intra-cluster correlation. Country stratification in the Cox model corrected for confounding by country-level baseline hazard differences. All predictors were selected a priori on theoretical grounds to minimise data-driven model specification bias. Missing data on birth weight, early breastfeeding initiation, and religion were handled using complete-case analysis, which under a missing-at-random assumption is unlikely to materially distort parameter estimates. Residual confounding by unmeasured variables, including breastfeeding intention, partner support, lactation counselling quality, and formula marketing exposure, cannot be excluded and is acknowledged as an inherent limitation of the observational design.

### Ethical considerations

Permission to access the DHS datasets from all 16 countries was obtained from the DHS Program following a formal data request. The original surveys were approved by the national ethics review committees and the ICF Institutional Review Board (IRB No. FWA00000845), with informed consent obtained from all participants. This study constituted a secondary analysis of deidentified, publicly available data; no additional ethical approval was needed. The data are available at https://dhsprogram.com/.

## Results

### Characteristics of the study sample

The pooled analytical sample comprised 20,085 infants aged 0–5 months from 16 SSA countries (DHS, 2019–2024). Overall, 75.74% (*n* = 15,212) of the infants were exclusively breastfed, whereas 24.26% (*n* = 4,871) were not (Table 2). The overall incidence rate of EBF cessation was 0.093 per person-month (95% CI: 0.090–0.095) across 51,047 person-months of observation (Table 5).

**Table 2:** Definition and measurement of individual-level predictor variables

No.	Variable	Definition and Measurement
1	Maternal age group	Age of the mother at survey, categorized as: 15–19, 20–34, and 35–49 years
2	Place of residence	Type of area of residence: Urban or Rural
3	Education level	Highest level of education attained: No education, Primary, Secondary, or Higher
4	Marital status	Current marital status: Never married, Married/in union, or Formerly married
5	Employment status	Maternal employment status at survey: Not working or Working
6	Wealth index	Household wealth status derived from the DHS composite wealth index: Poor, Middle, or Rich
7	Parity (children ever born)	Total children ever born: One, Two, Three, or Four or more
8	Media exposure	Exposure to any mass media (radio, newspaper, or television): No or Yes
9	Type of birth	Singleton or Multiple birth
10	Sex of child	Male or Female
11	Birth weight	Measured birth weight categorized as: Low (< 2,500 g), Normal (2,500–3,999 g), or High (≥ 4,000 g)
12	Early initiation of breastfeeding	Timing of initiation after delivery: Within 1 h or After 1 h of birth
13	Antenatal care visits	Number of ANC visits during most recent pregnancy: < 8 visits or ≥ 8 visits
14	Religion	Religious affiliation: Christian, Muslim, Other religion, or No religion

*ANC* Antenatal care, *DHS* Demographic and Health Survey

### Prevalence of nonexclusive breastfeeding by country

**Fig. 2:**
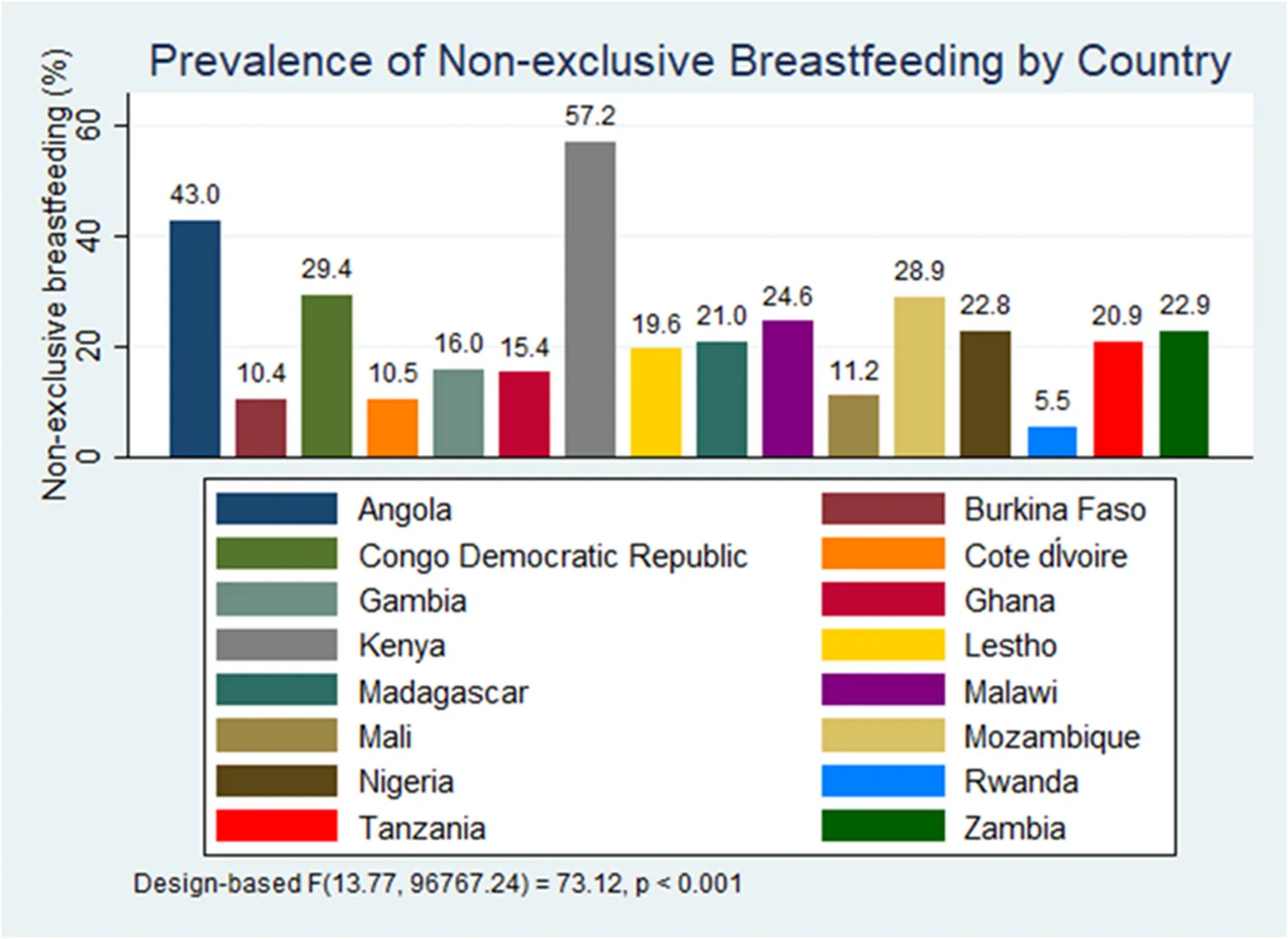
Prevalence of nonexclusive breastfeeding among infants aged 0–5 months, by country — DHS 2019–2024

Figure 2 illustrates the country-specific prevalence of non-EBF. Substantial variations were observed (p < 0.001). The highest prevalence was observed in Kenya (57.21%), followed by Angola (43.05%) and Congo DR (29.41%). Comparable levels were observed in Mozambique (28.91%), Malawi (24.64%), Zambia (22.88%), and Nigeria (22.78%). Lower prevalence estimates were recorded in Madagascar (21.04%), Tanzania (20.88%), and Lesotho (19.61%). The lowest prevalence rates were observed in Rwanda (5.51%), Burkina Faso (10.42%), Côte d’Ivoire (10.54%), and Mali (11.17%).

### Exclusive breastfeeding by individual-level characteristics

Table 3 presents the distribution of EBF according to individual-level background characteristics. Early initiation of breastfeeding was significantly associated with EBF status (*p* < 0.001); infants whose mothers initiated breastfeeding within one hour of birth had a higher EBF incidence (80.11%) than those who breastfed after one hour (70.54%). Maternal education was significantly associated with EBF (*p* < 0.001), with the highest prevalence observed among women with no formal education (79.33%) and lower but comparable proportions among those with primary (73.58%), secondary (74.94%), and higher education (73.34%). Religion (*p* < 0.001), marital status (*p* = 0.001), the household wealth index (*p* < 0.001), and media exposure (*p* = 0.020) were also significantly associated with EBF. Maternal age, place of residence, employment status, parity, type of birth, child sex, birth weight, and ANC visits were not significantly associated with EBF status in the bivariate analysis. Missing data on birth weight, early breastfeeding initiation, and religion accounted for 10–20% of observations for those variables; complete-case analysis was used throughout, as missingness was unlikely to be differential with respect to EBF status.

**Table 3:** Distribution of percentages of exclusive breastfeeding among infants aged 0–5 months by individual-level background characteristics, sub-Saharan Africa DHS 2019–2024 (*N* = 20,085)

Background Characteristics	EBF: Yes, *n* (%)	EBF: No, *n* (%)	*p* value
Maternal age group			0.695
15–19 years	2,052 (75.30)	673 (24.70)	
20–34 years	10,601 (75.97)	3,353 (24.03)	
35–49 years	2,560 (75.17)	846 (24.83)	
Place of residence			0.446
Urban	4,926 (75.23)	1,622 (24.77)	
Rural	10,286 (75.99)	3,250 (24.01)	
Maternal education level			< 0.001
No education	4,778 (79.33)	1,245 (20.67)	
Primary	4,595 (73.58)	1,650 (26.42)	
Secondary	5,001 (74.94)	1,672 (25.06)	
Higher	838 (73.34)	304 (26.66)	
Marital status			0.001
Never married	1,385 (72.15)	535 (27.85)	
Married/in union	13,184 (76.43)	4,067 (23.57)	
Formerly married	644 (70.54)	269 (29.46)	
Household wealth index			< 0.001
Poor	6,723 (74.03)	2,359 (25.97)	
Middle	3,084 (75.97)	975 (24.03)	
Rich	5,406 (77.86)	1,537 (22.14)	
Employment status			0.787
Not working	8,116 (75.85)	2,584 (24.15)	
Working	7,097 (75.63)	2,287 (24.37)	
Parity (children ever born)			0.815
One	3,717 (75.87)	1,182 (24.13)	
Two	3,133 (76.26)	975 (23.74)	
Three	2,502 (75.97)	792 (24.03)	
Four or more	5,860 (75.30)	1,922 (24.70)	
Media exposure			0.02
Not exposed	10,467 (75.13)	3,465 (24.87)	
Exposed	4,745 (77.14)	1,406 (22.86)	
Type of birth			0.336
Singleton	14,978 (75.79)	4,784 (24.21)	
Multiple	234 (72.84)	87 (27.16)	
Sex of child			0.78
Male	7,712 (75.85)	2,456 (24.15)	
Female	7,500 (75.64)	2,416 (24.36)	
Birth weight			0.798
Low (< 2,500 g)	4,686 (79.52)	1,207 (20.48)	
Normal (2,500–3,999 g)	8,302 (79.08)	2,196 (20.92)	
High (≥ 4,000 g)	1,838 (78.79)	495 (21.21)	
Early initiation of breastfeeding			< 0.001
After 1 h of birth	6,459 (70.54)	2,698 (29.46)	
Within 1 h of birth	8,753 (80.11)	2,173 (19.89)	
Antenatal care visits			0.993
< 8 visits	14,207 (75.80)	4,535 (24.20)	
≥ 8 visits (WHO recommended)	922 (75.82)	294 (24.18)	
Religion			< 0.001
Christian	13,602 (75.99)	4,298 (24.01)	
Muslim	499 (72.66)	188 (27.34)	
Other religion	70 (62.08)	43 (37.92)	
No religion	80 (57.61)	59 (42.39)	

EBF: exclusive breastfeeding. p values from design-adjusted Pearson’s chi-square tests. Survey weights were applied throughout the study

### Exclusive breastfeeding by community-level characteristics

Table 4 presents the EBF distribution by community-level characteristics. Community education level was significantly associated with EBF (*p* < 0.001), with the highest prevalence in communities with a medium level of education (78.35%) and the lowest in communities with a high level of education (72.05%). The community wealth level was also significant (*p* = 0.030), being highest in medium-wealth communities (77.17%) and lowest in high-wealth communities (74.27%). Community media exposure (*p* = 0.394) and community employment level (*p* = 0.364) were not significantly associated with EBF.

**Table 4:** Distribution of percentage of exclusive breastfeeding by community-level characteristics, sub-Saharan Africa DHS 2019–2024 (*N* = 20,085)

Background Characteristics	EBF: Yes, *n* (%)	EBF: No, *n* (%)	*p* value
Community wealth level			0.03
Low	4,752 (75.86)	1,512 (24.14)	
Medium	5,230 (77.17)	1,547 (22.83)	
High	5,230 (74.27)	1,812 (25.73)	
Community education level			< 0.001
Low	5,028 (76.89)	1,511 (23.11)	
Medium	5,294 (78.35)	1,463 (21.65)	
High	4,890 (72.05)	1,897 (27.95)	
Community media exposure			0.394
Low	4,833 (74.89)	1,620 (25.11)	
Medium	5,383 (76.40)	1,663 (23.60)	
High	4,996 (75.88)	1,588 (24.12)	
Community employment level			0.364
Low	5,198 (75.11)	1,723 (24.89)	
Medium	4,951 (76.66)	1,507 (23.34)	
High	5,063 (75.52)	1,641 (24.48)	

*EBF* exclusive breastfeeding. p values were obtained from design-adjusted Pearson chi-square tests. Survey weights applied throughout

### Incidence of exclusive breastfeeding cessation by country

Table 5 presents the country-specific incidence rates of EBF cessation. Kenya had the highest incidence rate (0.195 per person-month; 95% CI: 0.183–0.208), followed by Angola (0.173; 95% CI: 0.159–0.188) and Congo DR (0.108; 95% CI: 0.100–0.116). Incidence rates close to the overall pooled estimate were observed in Zambia (0.092), Tanzania (0.089), Nigeria (0.087), and Madagascar (0.087). The lowest rates were recorded in Rwanda (0.020; 95% CI: 0.014–0.027), Côte d’Ivoire (0.042; 95% CI: 0.035–0.050), and Burkina Faso (0.043; 95% CI: 0.036–0.051).

**Table 5:** Country-specific incidence rates of exclusive breastfeeding cessation among infants aged 0–5 months, sub-Saharan Africa DHS 2019–2024

Country	*n* (weighted)	Person-time (months)	Failures, *n*	Incidence rate	95% CI
Angola	1,262	3,200	553	0.173	[0.159, 0.188]
Burkina Faso	1,254	2,823	120	0.043	[0.036, 0.051]
Congo DR	2,277	6,489	701	0.108	[0.100, 0.116]
Côte d’Ivoire	985	2,853	119	0.042	[0.035, 0.050]
Gambia	897	2,446	131	0.054	[0.045, 0.064]
Ghana	828	2,236	113	0.051	[0.042, 0.061]
Kenya	1,711	4,861	948	0.195	[0.183, 0.208]
Lesotho	258	707	57	0.081	[0.062, 0.105]
Madagascar	1,262	3,099	270	0.087	[0.077, 0.098]
Malawi	1,179	2,976	263	0.088	[0.078, 0.100]
Mali	1,717	3,832	178	0.046	[0.040, 0.054]
Mozambique	997	2,361	242	0.102	[0.090, 0.116]
Nigeria	2,727	6,673	583	0.087	[0.081, 0.095]
Rwanda	781	1,870	37	0.02	[0.014, 0.027]
Tanzania	1,105	2,647	236	0.089	[0.078, 0.101]
Zambia	844	1,974	181	0.092	[0.079, 0.106]
Total	20,085	51,047	4,732	0.093	[0.090, 0.095]

*CI* Confidence interval. The incidence rates were expressed as events per person-month of observation

### Kaplan–Meier survival probabilities of exclusive breastfeeding by maternal and country characteristics

**Fig. 3:**
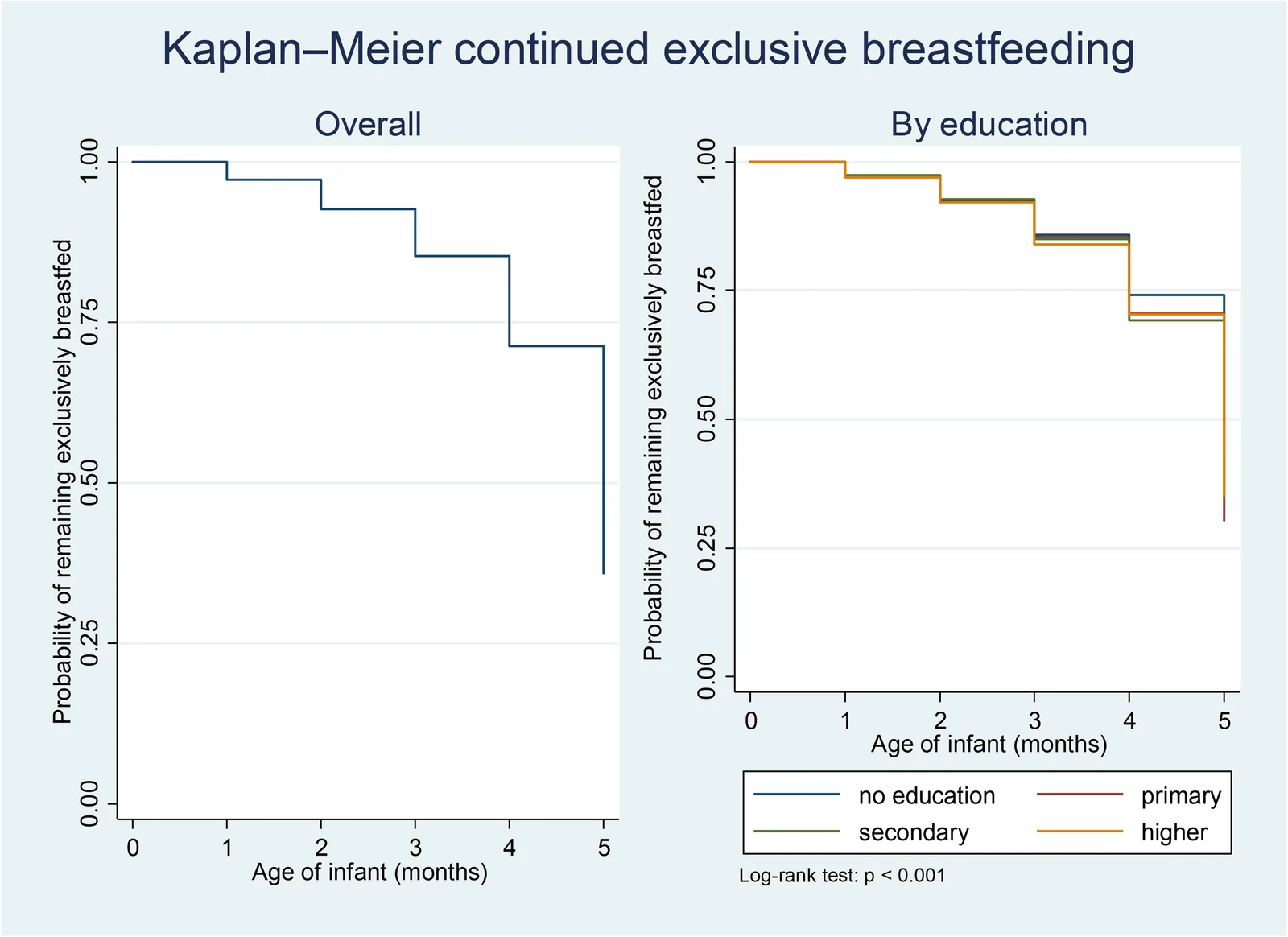
Kaplan–Meier survival curves for exclusive breastfeeding, overall and by maternal education level

**Fig. 4:**
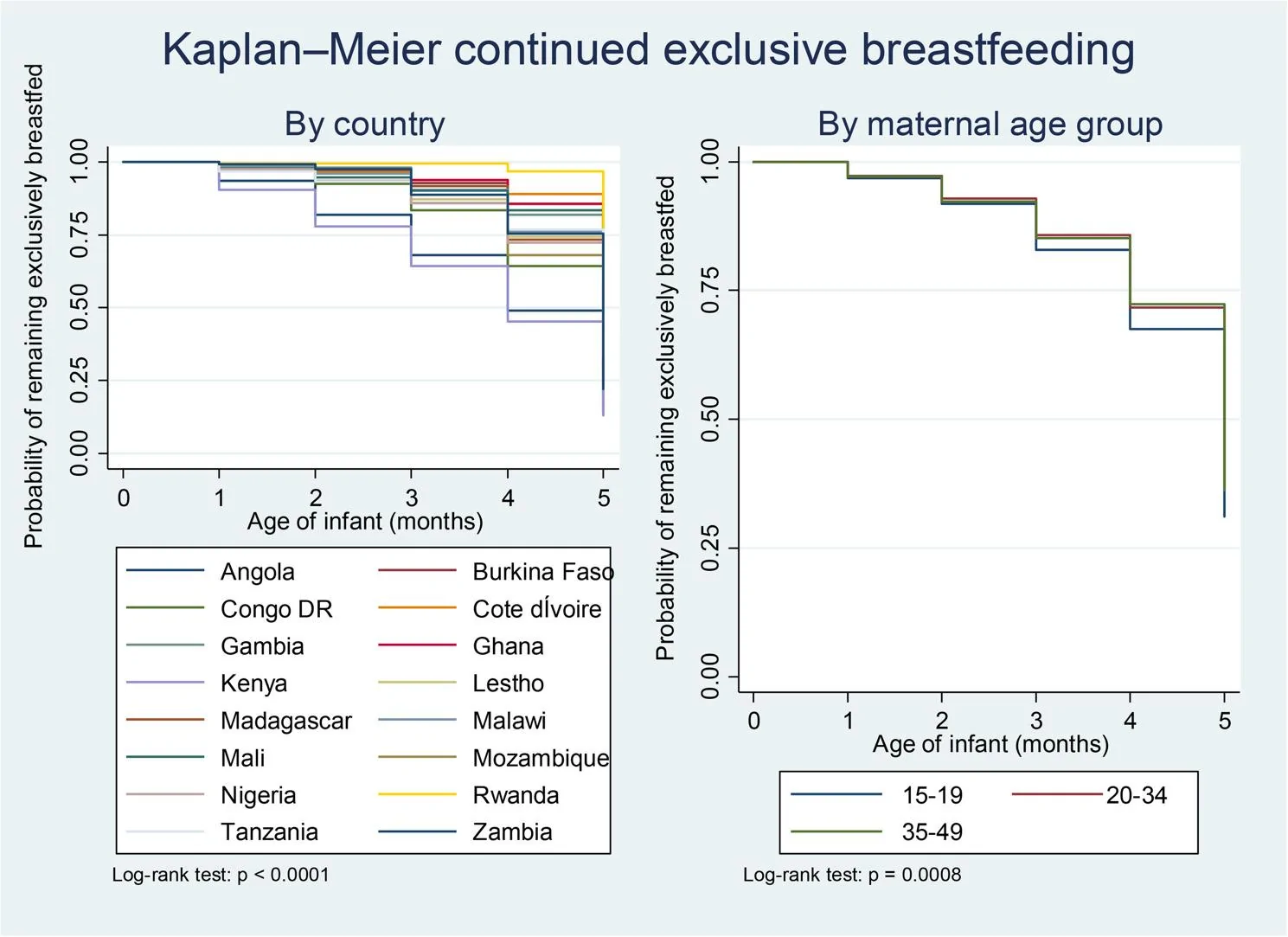
Kaplan–Meier survival curves for exclusive breastfeeding by country and maternal age group

The overall survival probability of remaining exclusively breastfed declined from 0.971 at month 1 to 0.926 at month 2, 0.853 at month 3, 0.713 at month 4, and 0.360 at month 5 (Fig. 3). A consistent decline was observed in all maternal education strata. By month 5, the survival probability was highest among mothers with no education (0.432) and lowest among those with primary education (0.305). Stratified by country, Rwanda maintained the highest survival (0.994 through month 3, declining to 0.760 by month 5), whereas Kenya (0.130) and Angola (0.143) recorded the lowest survival probabilities at month 5 (Fig. 4). By maternal age group, survival probabilities were similar at month 1 (range: 0.969–0.972) but diverged by month 5: mothers aged 20–34 years had the highest probability (0.368), whereas the youngest mothers (15–19 years) had the lowest probability (0.312).

### Kaplan–Meier survival analysis of exclusive breastfeeding duration by early initiation and birthweight

**Fig. 5:**
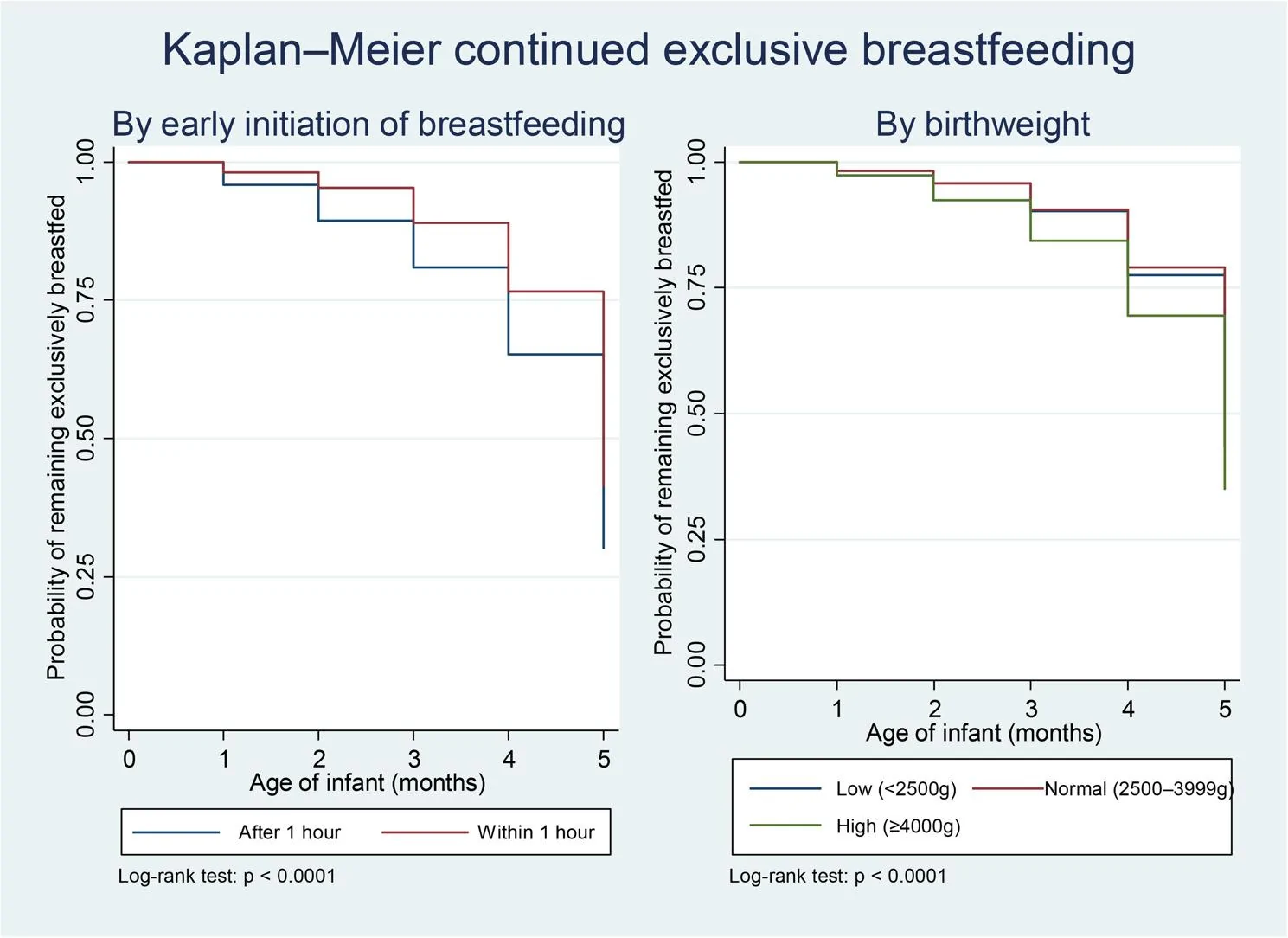
Kaplan–Meier survival curves for exclusive breastfeeding by birth weight category

Kaplan–Meier curves stratified by timing of breastfeeding initiation and birth weight category are presented in Fig. 5. Infants whose mothers-initiated breastfeeding within one hour of birth consistently maintained higher probabilities of continued exclusive breastfeeding throughout the follow-up period (survival probability at month 5: 0.750) compared with those whose initiation was delayed beyond one hour (0.280), with the survival curves diverging substantially from month 3 onwards. The log-rank test confirmed that this difference was statistically significant (*p* < 0.0001), consistent with the adjusted Cox model findings. Stratification by birth weight revealed that infants with normal birth weight (2500–3999 g) maintained the highest and most stable survival trajectory, with a probability of 0.750 at month 5. Infants with low birth weight (< 2500 g) followed a closely similar trajectory (0.750 at month 5), with modest declines observed at months 2 and 3 relative to the normal weight group. In contrast, infants with high birth weight (≥ 4000 g) demonstrated an initially comparable survival trajectory through month 4 but exhibited a pronounced late decline, with the survival probability falling sharply to approximately 0.380 by month 5, a pattern consistent with early supplementation or perceived milk insufficiency often reported among mothers of macrosomic infants. The log-rank test for birth weight strata was also statistically significant (*p* < 0.0001), corroborating birth weight as an independent predictor of EBF cessation in the adjusted model.

### Restricted mean survival time

As the Kaplan–Meier survival curve did not cross the 0.50 probability threshold in any of the 16 countries within the observation window, the conventional median survival time was non-estimable throughout, reflecting the predominantly high EBF retention in the pooled sample. The restricted mean survival time (RMST), defined as the area under the survival curve from month 0 to month 5, was therefore computed as the appropriate alternative summary measure of EBF duration. The pooled RMST was 4.46 person-months (95% CI: 4.45–4.48) out of a maximum of 5 months, indicating that infants across the 16 countries were exclusively breastfed for approximately 89% of the recommended duration on average. Substantial country-level variation was observed, with RMST ranging from 3.78 person-months (95% CI: 3.71–3.85) in Kenya to 4.95 person-months (95% CI: 4.92–4.98) in Rwanda, mirroring the country-specific cessation incidence heterogeneity reported above.

### Determinants of exclusive breastfeeding cessation: cox proportional hazards model

The crude and adjusted hazard ratios from the multivariable Cox proportional hazards model are presented across two tables for clarity: Table 6 presents individual-level factors and Table 7 presents community-level factors; both derive from a single model in which all variables were entered simultaneously. In the adjusted model, five independent predictors of EBF cessation were identified. Higher maternal education was associated with a significantly lower hazard of cessation compared to no education (aHR: 0.82; 95% CI: 0.69–0.98), and Muslim mothers had a 27% lower adjusted hazard of cessation than Christian mothers (aHR: 0.73; 95% CI: 0.60–0.88). Early initiation of breastfeeding within one hour of birth was independently associated with an 11% reduction in the hazard of EBF cessation compared to initiation after one hour (aHR: 0.89; 95% CI: 0.84–0.94).

**Table 6:** Crude and adjusted hazard ratios for individual-level determinants of exclusive breastfeeding cessation: Sub-Saharan Africa DHS 2019–2024 (*N* = 20,085)

Variable	*n*	%	cHR (95% CI)	aHR (95% CI)
Maternal age group				
15–19 years (Ref)	2,726	13.57	1.00 (ref)	1.00 (ref)
20–34 years	13,953	69.47	0.87** (0.81–0.93)	0.91 (0.83–1.01)
35–49 years	3,406	16.96	0.88** (0.81–0.96)	0.96 (0.85–1.09)
Maternal education level				
No education (Ref)	6,022	29.98	1.00 (ref)	1.00 (ref)
Primary	6,247	31.1	1.19** (1.11–1.28)	0.99 (0.91–1.07)
Secondary	6,674	33.23	1.16** (1.08–1.25)	0.98 (0.90–1.07)
Higher	1,142	5.69	1.16* (1.01–1.32)	0.82* (0.69–0.98)
Marital status				
Never married (Ref)	1,921	9.57	1.00 (ref)	1.00 (ref)
Married/in union	17,250	85.89	0.85** (0.78–0.91)	0.98 (0.89–1.08)
Formerly married	912	4.54	0.96 (0.86–1.09)	0.90 (0.77–1.05)
Household wealth index				
Poor (Ref)	9,082	45.22	1.00 (ref)	1.00 (ref)
Middle	4,059	20.21	0.92* (0.86–0.98)	0.96 (0.89–1.04)
Rich	6,944	34.57	0.88** (0.82–0.93)	0.96 (0.87–1.05)
Employment status				
Not working (Ref)	10,702	53.28	1.00 (ref)	1.00 (ref)
Working	9,383	46.72	0.85** (0.81–0.90)	0.98 (0.92–1.04)
Religion				
Christian (Ref)	17,900	95.02	1.00 (ref)	1.00 (ref)
Muslim	686	3.64	1.18** (1.05–1.32)	0.73** (0.60–0.88)
Other religion	113	0.6	1.34* (1.01–1.79)	1.12 (0.84–1.51)
No religion	138	0.73	1.97** (1.53–2.55)	1.13 (0.88–1.44)
Parity				
One (Ref)	4,900	24.4	1.00 (ref)	1.00 (ref)
Two	4,109	20.46	1.00 (0.93–1.08)	1.02 (0.93–1.12)
Three	3,294	16.4	0.95 (0.88–1.03)	1.04 (0.94–1.16)
Four or more	7,782	38.74	0.98 (0.92–1.04)	1.03 (0.93–1.13)
Birth weight				
Low, < 2,500 g (Ref)	1,281	6.65	1.00 (ref)	1.00 (ref)
Normal, 2,500–3,999 g	10,020	52.02	0.98 (0.87–1.10)	1.03 (0.91–1.16)
High, ≥ 4,000 g	7,961	41.33	1.34** (1.19–1.51)	1.22** (1.07–1.39)
Early initiation of breastfeeding				
After 1 h of birth (Ref)	9,157	45.59	1.00 (ref)	1.00 (ref)
Within 1 h of birth	10,928	54.41	0.68** (0.64–0.72)	0.89** (0.84–0.94)
Media exposure				
Not exposed (Ref)	13,932	69.36	1.00 (ref)	1.00 (ref)
Exposed	6,153	30.64	0.91** (0.86–0.97)	1.04 (0.97–1.12)
ANC visits				
< 8 visits (Ref)	18,744	93.91	1.00 (ref)	1.00 (ref)
≥ 8 visits	1,216	6.09	0.96 (0.86–1.06)	1.16* (1.03–1.30)

*cHR* crude hazard ratio, *aHR* adjusted hazard ratio, *CI* confidence interval, *ANC* antenatal care, *Ref* reference category. The model was stratified by country, and clustering was adjusted using robust standard errors. †The crude hazard ratio for community wealth “High” was not estimable in the unadjusted model due to a convergence issue; the adjusted estimate is reported. *p < 0.05; **p < 0.01. Variables with missing data excluded under complete-case analysis: religion (*n* = 1,248; 6.21%), birth weight (*n* = 823; 4.10%), and ANC visits (*n* = 125; 0.62%). All other variables had complete data for the full analytical sample (*N* = 20,085). Missingness was considered unlikely to be differential with respect to EBF status

Conversely, infants with high birth weight (≥ 4,000 g) had a 22% greater hazard of cessation relative to low birth weight infants (aHR: 1.22; 95% CI: 1.07–1.39), and attending eight or more ANC visits was associated with a 16% greater hazard of cessation compared to fewer visits (aHR: 1.16; 95% CI: 1.03–1.30). Variables that were significant in crude models, including maternal age, marital status, household wealth, employment status, and media exposure, lost statistical significance after adjustment, suggesting their apparent effects were largely explained by confounding. The crude and adjusted hazard ratios from the multivariable Cox proportional hazards model are presented across two tables for clarity: Table 6 presents individual-level factors and Table 7 presents community-level factors. Both tables derive from a single model in which all variables were entered simultaneously.

**Table 7:** Crude and adjusted hazard ratios for community level determinants of exclusive breastfeeding cessation: sub-Saharan Africa DHS 2019–2024 (*N* = 20,085)

Variable	*n*	%	cHR (95% CI)	aHR (95% CI)
Place of residence				
Urban (Ref)	6,549	32.61	1.00 (ref)	1.00 (ref)
Rural	13,536	67.39	1.00 (0.94–1.06)	1.03 (0.95–1.12)
Community wealth level				
Low (Ref)	6,264	31.19	1.00 (ref)	1.00 (ref)
Medium	6,779	33.75	0.92 (0.86–0.99)	0.97 (0.90–1.05)
High	7,042	35.06	1.00 (0.93–1.07)	1.00 (0.92–1.09)
Community education level				
Low (Ref)	6,539	32.56	1.00 (ref)	1.00 (ref)
Medium	6,758	33.65	0.95 (0.88–1.01)	0.98 (0.91–1.05)
High	6,787	33.79	1.15** (1.07–1.23)	1.04 (0.96–1.14)
Community media exposure				
Low (Ref)	6,453	32.13	1.00 (ref)	1.00 (ref)
Medium	7,048	35.09	0.94* (0.88–1.00)	1.03 (0.96–1.11)
High	6,584	32.78	0.95 (0.89–1.02)	1.00 (0.93–1.09)
Community employment level				
Low (Ref)	6,923	34.47	1.00 (ref)	1.00 (ref)
Medium	6,458	32.15	0.92 (0.87–0.99)	1.00 (0.94–1.08)
High	6,704	33.38	0.93 (0.87–1.00)	0.96 (0.89–1.03)

### Community-level determinants of exclusive breastfeeding cessation

For the community-level factors (Table 7), most variables that appeared significant in crude models lost statistical significance after adjustment, suggesting limited independent community-level influence on EBF cessation once individual-level characteristics are accounted for. Place of residence showed no significant association with EBF cessation in either crude or adjusted models, indicating that rural-urban differences in EBF duration were largely explained by individual-level compositional factors rather than residence per se.

Community education level was the only community-level variable with a significant crude association, women in high community education settings had a 15% higher hazard of cessation compared to low community education settings (cHR: 1.15, 95% CI: 1.07–1.23) — but this too became non-significant after adjustment (aHR: 1.04, 95% CI: 0.96–1.14). Community wealth, community media exposure, and community employment level showed no significant associations in adjusted models, suggesting these aggregate contextual factors do not independently shape EBF duration beyond what is captured by individual-level determinants.

Overall, the results from both tables point to individual-level factors, particularly early initiation of breastfeeding, high infant birth weight, higher maternal education, Muslim religious affiliation, and adequate ANC attendance, as the primary independent determinants of EBF cessation in this pooled sub-Saharan African sample, with community-level factors playing a minimal independent role.

### Parametric Gompertz regression analysis

The Gompertz parametric model confirmed the robustness of the Cox model findings, with a consistent direction and significance of the associations across both models (Supplementary Table S1). Eleven countries presented positive and statistically significant Gompertz shape parameters (γ), indicating an increasing hazard of EBF cessation over time: Rwanda (γ = 1.299; *p* = 0.015), Madagascar (γ = 0.774), Zambia (γ = 0.757), Malawi (γ = 0.653), Gambia (γ = 0.588), Ghana (γ = 0.506), Kenya (γ = 0.451), Côte d’Ivoire (γ = 0.401), Mozambique (γ = 0.345), Congo DR (γ = 0.257), and Nigeria (γ = 0.214; all *p* < 0.001 or *p* = 0.001). In contrast, Angola (reference), Burkina Faso (γ = 0.135; *p* = 0.147), Lesotho (γ = 0.183; *p* = 0.156), and Mali (γ = −0.034; *p* = 0.611) showed nonsignificant γ estimates, suggesting no clear monotonic change in the hazard over time in these settings (Table 8).

**Table 8:** Country-specific Gompertz shape parameters (γ) for cessation of exclusive breastfeeding, Sub-Saharan Africa DHS 2019–2024

Country	γ (SE)	*p* value	95% CI
Angola (Reference)	—	—	—
Burkina Faso	0.135	0.147	[− 0.047, 0.318]
Congo DR	0.257	< 0.001	[0.164, 0.350]
Côte d’Ivoire	0.401	0.001	[0.154, 0.648]
Gambia	0.588	< 0.001	[0.349, 0.828]
Ghana	0.506	< 0.001	[0.250, 0.761]
Kenya	0.451	< 0.001	[0.244, 0.658]
Lesotho	0.183	0.156	[− 0.070, 0.436]
Madagascar	0.774	< 0.001	[0.582, 0.966]
Malawi	0.653	< 0.001	[0.466, 0.841]
Mali	−0.034	0.611	[− 0.163, 0.096]
Mozambique	0.345	< 0.001	[0.187, 0.502]
Nigeria	0.214	< 0.001	[0.110, 0.317]
Rwanda	1.299	0.015	[0.251, 2.347]
Tanzania (omitted)	0	—	—
Zambia	0.757	< 0.001	[0.538, 0.976]

*γ* Gompertz shape parameter. A positive γ indicates an increasing hazard of EBF over time. Angola was the reference country. *SE* standard error, *CI* confidence interval. *p < 0.05; **p < 0.01; ***p < 0.001

## Discussion

This study examined the timing and determinants of exclusive breastfeeding cessation among infants aged 0–5 months across 16 sub-Saharan African countries using pooled DHS data and survival analysis methods. Consistent with previous evidence, EBF cessation was influenced predominantly by individual-level characteristics, including maternal education, religious affiliation, timing of breastfeeding initiation, birth weight, and antenatal care attendance. However, the principal contribution of this study extends beyond identifying determinants of cessation. By incorporating country-specific Gompertz parametric models, we were able to characterize the temporal trajectories of cessation risk across countries, providing evidence not only on who is at greater risk of discontinuing EBF, but also on when cessation risk accelerates. The findings reveal substantial heterogeneity in the evolution of cessation hazards across SSA, highlighting the importance of aligning breastfeeding interventions with country-specific risk trajectories rather than relying solely on prevalence estimates or universal implementation schedules.

The overall EBF prevalence of 75.74% and pooled cessation incidence rate of 0.093 per person-month were higher than the 41%–49% prevalence estimates reported in recent multi-country cross-sectional analyses from SSA [11, 12]. This difference is largely methodological rather than contradictory. Our analysis was restricted to last-born infants aged 0–5 months, and EBF status was assessed using 24-hour dietary recall at the time of the survey. Furthermore, the survival framework accounted for right-censoring and focused specifically on age-eligible infants during the recommended six-month breastfeeding period. These methodological features are likely to yield higher estimates than retrospective or age-aggregated population approaches commonly used in cross-sectional studies [15, 16].

Despite these relatively favourable estimates, the findings confirm that early cessation of EBF remains a substantial and preventable public health challenge across SSA. This is particularly important in the context of the World Health Assembly’s revised target of achieving at least 60% EBF coverage by 2030 and Sustainable Development Goal (SDG) 2.2, which seeks to eliminate all forms of malnutrition [7, 8]. Accelerating progress toward these targets will require sustained investment in evidence-based interventions, including full implementation of the Baby-Friendly Hospital Initiative (BFHI), stronger enforcement of the International Code of Marketing of Breast-milk Substitutes, and expansion of maternity protection policies that enable women to continue breastfeeding during the first six months of life.

Substantial heterogeneity in country-specific EBF cessation incidence was a central finding, with rates ranging from 0.020 per person-month in Rwanda to 0.195 per person-month in Kenya. This range reflects the diversity of structural, cultural, and health system contexts across SSA and underscores the inadequacy of region-wide uniform policy responses. Kenya and Angola recorded the highest cessation rates, consistent with evidence that rapid urbanisation, increasing maternal labour force participation, inadequate maternity protection legislation, weak workplace breastfeeding policies, and heightened exposure to commercial infant formula marketing are significant drivers of early EBF cessation in these settings [11, 20, 21]. Both countries face particular challenges in translating national breastfeeding policy into practice at the facility and community levels [20]. Targeted responses in high-cessation settings should prioritise mandatory enforcement of paid maternity leave of at least 18 weeks as recommended by the International Labour Organization (ILO) [22, 23], the establishment of lactation rooms and flexible nursing breaks, and strengthened regulatory oversight of breast-milk substitute marketing in line with the International Code and subsequent World Health Assembly resolutions [24].

In contrast, Rwanda and Burkina Faso recorded the lowest cessation rates. Rwanda’s success reflects the integrated impact of its community-based IMCI programme, a network of approximately 45,000 community health workers (CHWs), and systematic BFHI implementation across maternity facilities [25, 26]. Burkina Faso demonstrates that sustained community-level peer counselling, as evidenced in the PROMISE-EBF trial, where CHW-delivered EBF promotion achieved 71% EBF in the intervention arm versus 9% in controls can maintain low cessation rates even in resource-constrained settings [27]. These high-performing countries offer replicable models for higher-cessation settings across SSA, centred on the twin pillars of facility-level BFHI and community-level CHW deployment.

The Kaplan–Meier analysis demonstrated that the probability of remaining exclusively breastfed declined from 0.971 at month 1 to 0.360 by month 5, with the steepest decline occurring between months three and five. This pattern is consistent with prior survival analyses of EBF duration in SSA [15, 16] and identifies the three-to-five-month period as a critical window of vulnerability. This timing converges with re-emergence of traditional practices of early complementary food introduction, declining maternal confidence in milk sufficiency particularly among younger and primiparous mothers and pressure to return to work or agricultural activities [5, 28].

The Gompertz sensitivity analysis extended these findings by characterising the temporal shape of cessation hazard within each country. Eleven of the 16 countries exhibited significantly positive γ estimates, indicating that cessation risk accelerates over time rather than remaining constant or declining. The steepest acceleration was observed in Rwanda (γ = 1.299; *p* = 0.015), a pattern that most plausibly reflects a ceiling effect: Rwanda’s strong breastfeeding infrastructure sustains EBF effectively in the earliest months, but the small proportion of mothers who do cease tend to do so with increasing probability as infants approach the five-month mark, possibly as cultural pressures toward complementary food introduction intensify or as CHW contact frequency declines in later infancy. Madagascar (γ = 0.774), Zambia (γ = 0.757), Malawi (γ = 0.653), and Gambia (γ = 0.588) showed similarly steep acceleration, suggesting that postnatal support systems in these countries, while effective in early infancy, fail to sustain protective effects through the critical three-to-five-month window. Ghana, Kenya, Côte d’Ivoire, Mozambique, Congo DR, and Nigeria showed more gradual but still significantly increasing hazards.

By contrast, Angola, Burkina Faso, Lesotho, and Mali showed nonsignificant γ estimates, indicating no statistically detectable monotonic change in cessation hazard over the observation window. These patterns are programmatically distinct. Angola’s persistently elevated cessation incidence rate (0.173 per person-month) despite a flat temporal trajectory implies that cessation risk is uniformly high from the earliest weeks of life, pointing to structural and commercial barriers that operate immediately postpartum rather than accumulating over time. Burkina Faso and Mali, by contrast, combine low overall incidence with flat trajectories, reflecting health systems that effectively and consistently suppress cessation risk across the entire 0–5 month period [27, 29]. Lesotho’s nonsignificant γ warrants cautious interpretation given its small country-specific sample (*n* = 707 person-months), which limits statistical power to detect a temporal trend even if one exists.

These temporal patterns carry concrete programmatic implications. Countries with steeply accelerating hazards, particularly Madagascar, Zambia, Malawi, and Gambia should intensify breastfeeding support contacts specifically between months two and five through scheduled CHW home visits, peer counsellor deployment, and mHealth reminder systems [27, 30]. For Angola, where risk is uniformly elevated from birth, interventions must begin immediately postpartum and sustain high intensity from the first days of life, including at least two facility-based postnatal visits within the first week as recommended by WHO [31, 32] and robust CHW contact in the neonatal period [33]. The fundamental implication across all settings is that uniform, age-undifferentiated breastfeeding promotion strategies are insufficient: the temporal profile of cessation risk within each country should inform when, not only for whom, postnatal support resources are deployed.

Higher maternal education specifically tertiary or higher education relative to no formal education was independently protective against EBF cessation (aHR = 0.82; 95% CI: 0.69–0.98). The crude analysis revealed a paradoxical pattern in which all education levels were associated with greater cessation hazard relative to no education, a pattern well-documented across SSA and attributable to confounding by formal employment, urban residence, and greater exposure to commercial infant formula marketing among educated women [9, 11]. Multivariable adjustment disentangled these confounders, revealing that higher education confers protection most plausibly through pathways of greater health literacy, enhanced autonomy in infant feeding decision-making, and stronger engagement with evidence-based breastfeeding counselling [6, 9]. That the protective effect was statistically significant only at the tertiary level suggests a threshold relationship rather than a linear dose-response. Breastfeeding promotion programs should recognise that lower-educated women are not inherently protected their higher crude EBF rates may partly reflect limited access to formula substitutes rather than active breastfeeding commitment while ensuring that formally employed, educated women have access to structural workplace supports, including legislated maternity leave of at least 18 weeks, dedicated lactation spaces, and return-to-work breastfeeding counselling [6, 22, 23].

Muslim religious affiliation was the strongest protective predictor in the adjusted model (aHR = 0.73; 95% CI: 0.60–0.88). This finding is consistent with the normative guidance embedded in Islamic scripture specifically Surah Al-Baqarah (2:233), which instructs mothers to breastfeed for two complete years and with the documented role of Islamic normative frameworks as powerful cultural drivers of prolonged breastfeeding across Muslim-majority communities in SSA [18]. The striking reversal from the crude estimate (cHR = 1.18) to the adjusted estimate (aHR = 0.73) reflects confounding by country-level composition: Muslim-majority countries in the Sahel (Mali, Burkina Faso, Gambia) tend to have both higher EBF rates and distinct sociodemographic profiles relative to Christian-majority countries, generating apparent crude confounding that disappears after adjustment. These findings support faith-based engagement as a high-yield, low-cost, and culturally legitimate strategy for EBF promotion. National breastfeeding programs should actively partner with Islamic religious leaders, mosque networks, and faith-based health workers to disseminate breastfeeding promotion messaging grounded in religious normative frameworks [6, 18]. Similar engagement strategies should be explored with other religious communities.

Early initiation of breastfeeding within one hour of birth was associated with an 11% reduction in the adjusted hazard of EBF cessation (aHR = 0.89; 95% CI: 0.84–0.94). The biological mechanism is well established: early suckling and skin-to-skin contact stimulate oxytocin and prolactin release, establishing hormonal cascades that underpin sustained milk production, reinforce maternal breastfeeding confidence, and facilitate infant latching all of which reduce the probability of early cessation [34–36]. Early initiation also functions as a sentinel indicator of the quality of postnatal care and the breastfeeding-supportive environment at facility level. These findings provide quantitative support for universal scale-up of the BFHI across SSA maternity facilities, with priority given to consistent implementation of immediate skin-to-skin contact, avoidance of unnecessary mother-infant separation, and restriction of routine formula supplementation without clinical indication [37, 38]. Community-based birth preparedness programs should additionally incorporate counselling on early initiation for home deliveries, which remain prevalent in several high-cessation countries including Angola and Nigeria [39, 40], and skilled birth attendant training curricula should include competency-based modules on facilitating early breastfeeding initiation.

High birth weight (≥ 4,000 g) was associated with a 22% greater adjusted hazard of EBF cessation (aHR = 1.22; 95% CI: 1.07–1.39), a finding robust across both the Cox and Gompertz models. Four plausible mechanisms may underlie this association. First, macrosomic infants present with elevated caloric demands that may reinforce maternal perceptions of insufficient milk supply, one of the most commonly cited drivers of early cessation in SSA [10, 11]. Second, macrosomia is strongly associated with gestational diabetes mellitus and maternal obesity, both of which independently predict delayed lactogenesis II and greater likelihood of in-hospital formula supplementation. Third, in many SSA cultural contexts, a large infant may paradoxically reduce perceived urgency to sustain EBF, as birth weight is interpreted as a signal of adequate nutritional reserve. Fourth, clinical protocols for monitoring macrosomic neonates for hypoglycaemia may directly interrupt EBF establishment in settings where formula supplementation is used as a first-line intervention. Clinically, mothers of macrosomic infants should be proactively identified as a high-risk group for early cessation, with structured lactation support beginning immediately after delivery. Facility protocols for macrosomic newborn management should be reviewed to ensure glucose monitoring does not default to formula as a first-line intervention where frequent breastfeeding and close monitoring are clinically safe. Birth weight-stratified postnatal care pathways, with intensified lactation support contacts in the first two postpartum weeks, represent a practical and scalable response.

Attending eight or more ANC visits was associated with a 16% greater adjusted hazard of EBF cessation (aHR = 1.16; 95% CI: 1.03–1.30). This paradoxical finding most plausibly reflects health-related selection bias: women accumulating eight or more ANC contacts are disproportionately those with high-risk pregnancies including gestational diabetes, hypertensive disorders, and fetal growth abnormalities conditions that independently predict suboptimal lactation, delayed milk onset, and greater clinical pressure toward formula supplementation [41, 42]. This interpretation is consistent with the well-established sick-user phenomenon in observational studies of healthcare utilisation, whereby higher service contact reflects greater underlying clinical risk rather than causal harm from the service itself. Beyond selection bias, ANC contact frequency is fundamentally a measure of quantity rather than counselling quality or content fidelity.

Women with high ANC contact disproportionately concentrated in urban settings may simultaneously face greater occupational breastfeeding constraints, heightened clinic-based formula marketing exposure, and reduced access to community peer support networks [21, 43]. This finding should not be interpreted as evidence that ANC attendance is harmful to breastfeeding, nor as grounds to revisit WHO’s eight-contact recommendation. Rather, it delivers a clear programmatic message: the quality and fidelity of breastfeeding counselling delivered at each ANC contact is at least as important as, and likely more important than the number of contacts achieved. National ANC programmes should embed standardised, competency-assessed breastfeeding counselling modules at every contact, train providers in motivational interviewing to address formula feeding intentions, and develop structured referral pathways to lactation specialists and peer support groups for women identified as high-risk for early cessation during the antenatal period.

Collectively, these findings argue for the routine incorporation of survival analysis frameworks, including time-to-event modelling and Gompertz or Weibull parametric modelling into national nutrition monitoring and evaluation systems across SSA. Most national breastfeeding monitoring systems currently rely on cross-sectional prevalence estimates from household surveys, which obscure the temporal dynamics of cessation and preclude identification of age-specific vulnerability windows. The survival analysis approach demonstrated here enables programmes to pinpoint precisely when cessation risk peaks within the 0–5 month window, to allocate postnatal support resources to the periods of greatest need, and to evaluate time-targeted interventions through changes in country-specific γ estimates across successive survey rounds. The DHS Programme’s standardised data collection infrastructure provides an existing and scalable platform for this kind of monitoring [44], and national statistical offices and ministries of health should be supported to build the analytical capacity required to implement it routinely. Given the well-established causal role of EBF in reducing child mortality, preventing noncommunicable disease, and supporting cognitive development and its direct alignment with SDG 2.2, SDG 3, and SDG 10 [7, 8] investment in survival analysis-based breastfeeding monitoring represents a high-return, evidence-justified priority for global health funders and national governments [2, 6].

### Strengths and limitations

This study has several notable strengths. First, the pooled analytical sample of 20,830 last-born infants drawn from 16 nationally representative DHS surveys conducted between 2019 and 2024 is one of the largest and most geographically diverse assembled for a survival analysis of EBF cessation in SSA. The use of harmonised DHS Round 8 protocol data across all 16 countries ensures comparability of variable definitions, questionnaire content, and data collection procedures, strengthening the validity of the pooled and cross-country comparative analyses. Second, country stratification in the Cox model, combined with PSU-level robust standard errors, appropriately accounts for both country-level baseline hazard differences and intra-cluster correlation arising from the complex survey design. Third, the inclusion of community-level contextual variables extends the analysis beyond individual determinants, and the Gompertz parametric sensitivity analysis provides country-specific temporal intelligence on when cessation risk accelerates, a dimension rarely reported in multi-country EBF studies. Fourth, post-hoc power estimation confirms adequate power to detect hazard ratios of modest to moderate magnitude, and country-specific RMST reporting provides a clinically interpretable complement to the hazard-based findings.

This study is subject to several limitations. The cross-sectional current-status design means that cessation timing is approximated rather than directly observed, constituting interval censoring within monthly intervals and precluding the precision of prospective designs. EBF status assessed by 24-hour dietary recall captures point-in-time rather than habitual feeding behaviour; both sources of misclassification are expected to be non-differential and to bias estimates conservatively toward the null. Residual confounding by variables unavailable in the DHS, including breastfeeding intention, partner support, lactation counselling quality, postnatal care contact frequency, and formula marketing exposure cannot be excluded, and causal inference is precluded by the observational design. Missing data on birth weight (10–20% of observations) was addressed using complete-case analysis under a missing-at-random assumption, which may reduce precision for that predictor. Community-level variables derived by aggregating individual responses within enumeration areas represent compositional rather than purely contextual characteristics; their non-significance after adjustment should not be interpreted as definitive evidence of absent contextual effects, as a formal multilevel survival model with independent structural measures would be required to test this. Country-specific Gompertz shape parameters should be interpreted cautiously in smaller samples, particularly Lesotho (707 person-months). The pooled Cox model does not test for country-level effect modification, which is plausible given the substantial diversity across settings and warrants formal investigation in future research. Finally, findings are generalisable to last-born infants aged 0–5 months across comparable SSA settings within the 2019–2024 survey period and should not be extrapolated to non-SSA contexts or more recent timepoints not captured by the included surveys.

The unexpected positive association between attendance at eight or more ANC visits and greater EBF cessation hazard, contrary to the anticipated protective direction was not explored through formal sensitivity analyses such as restriction to low-risk pregnancies or stratification by parity and delivery complications, which would have been required to adequately disentangle health-related selection effects from any true ANC influence on breastfeeding behaviour. This association should therefore be interpreted with particular caution, and dedicated prospective studies with richer clinical data are needed to clarify the relationship between high ANC contact and breastfeeding outcomes.”

## Conclusions

Exclusive breastfeeding cessation remains a significant public health challenge across sub-Saharan Africa, with considerable variation in both the incidence and timing of cessation between countries. Individual-level factors, including maternal education, religious affiliation, early initiation of breastfeeding, birth weight, and antenatal care attendance, were important predictors of cessation, whereas community-level factors did not independently predict cessation after adjustment.

A key contribution of this study is the demonstration that cessation risk does not evolve uniformly across countries. Through the use of country-specific Gompertz models, this analysis provides novel evidence on the temporal dynamics of EBF cessation, identifying settings where the hazard of cessation accelerates rapidly during the first months of life. These findings offer an additional layer of programmatic intelligence beyond conventional prevalence estimates by indicating when intervention efforts are likely to have the greatest impact. Future breastfeeding programmes should therefore be designed not only around populations at risk but also around the timing of risk. Integrating breastfeeding counselling throughout antenatal and postnatal care, strengthening early initiation practices through Baby-Friendly Hospital Initiative strategies, and tailoring postnatal support according to country-specific cessation trajectories may substantially improve EBF retention and contribute to achieving global nutrition and child survival targets.

In the adjusted models, statistically significant associations were observed for individual-level factors only; no community-level variable reached significance after adjustment. However, this should not be interpreted as evidence that contextual factors are unimportant, as a formal multilevel survival model was not fitted and the community-level constructs were derived by aggregation of individual responses rather than from independent structural measures.

## Supplementary Information

Supplementary Material 1.

Supplementary Material 2.

## Data Availability

The datasets analyzed in this study are available upon request from the DHS Program website ( https://dhsprogram.com/). Data access requests are subject to the DHS program terms and conditions.

## Abbreviations

AIC: Akaike information criterion
ANC: antenatal care
aHR: adjusted hazard ratio
BFHI: Baby-Friendly Hospital Initiative
BIC: Bayesian information criterion
cHR: crude hazard ratio
CI: confidence interval
DHS: demographic and health survey
EA: enumeration area
EBF: exclusive breastfeeding
HR: hazard ratio
KR: children’s record
PSU: primary sampling unit
SSA: sub-Saharan Africa
VIF: variance inflation factor
WHO: World Health Organization
