# Supplementary material for: Determinants and temporal trajectories of exclusive breastfeeding cessation in 16 sub-Saharan African countries: a pooled multivariable survival analysis of the demographic and health survey (2019–2024)
Source: BMC Public Health. 2026 Jul 15;26:2896. doi: 10.1186/s12889-026-28431-9 (PMC13651175; doi:10.1186/s12889-026-28431-9)
Supplement: Supplementary file 1 [file 12889_2026_28431_MOESM1_ESM.docx]

**STROBE Statement — Checklist of items that should be included in reports of cross-sectional studies**

*Manuscript title: Determinants and Temporal Trajectories of Exclusive Breastfeeding Cessation in 16 Sub-Saharan African Countries: A Pooled Multivariable Survival Analysis of the Demographic and Health Survey (2019–2024)*

| **Section/Topic** | **Item No** | **Recommendation** | **Page No** |
| --- | --- | --- | --- |
| **Title and abstract** | | | |
|  | 1 | (a) Indicate the study's design with a commonly used term in the title or the abstract | Page 1 Design stated as "pooled multivariable survival analysis" in title and abstract |
|  |  | (b) Provide in the abstract an informative and balanced summary of what was done and what was found | Pages 1–2 Structured abstract: Background, Methods, Results, Conclusions |
| **Introduction** | | | |
| Background/rationale | 2 | Explain the scientific background and rationale for the investigation being reported | Pages 3–5 Background section; community-level pathways paragraph |
| Objectives | 3 | State specific objectives, including any prespecified hypotheses | Page 5 Final paragraph of Background |
| **Methods** | | | |
| Study design | 4 | Present key elements of study design early in the paper | Page 6 Data Source and Participant Selection subsections |
| Setting | 5 | Describe the setting, locations, and relevant dates, including periods of recruitment, exposure, follow-up, and data collection | Pages 6–7 Data Source: 16 SSA countries, DHS 2019–2024, Table 1 |
| Participants | 6 | (a) Give the eligibility criteria, and the sources and methods of selection of participants | Pages 6–7 Participant Selection: last-born infants aged 0–5 months, alive at survey; Figure 1 flow diagram |
| Variables | 7 | Clearly define all outcomes, exposures, predictors, potential confounders, and effect modifiers. Give diagnostic criteria, if applicable | Pages 7–9 Outcome Variable, Explanatory Variables, Community-level factors; Tables 2 |
| Data sources/measurement | 8* | For each variable of interest, give sources of data and details of methods of assessment (measurement). Describe comparability of assessment methods if there is more than one group | Pages 6–9 DHS KR files; 24-hour dietary recall for EBF; DHS variable b19 for age; Tables 2 |
| Bias | 9 | Describe any efforts to address potential sources of bias | Page 11 Bias subsection: survey weights, PSU clustering, country stratification, complete-case analysis, a priori variable selection |
| Study size | 10 | Explain how the study size was arrived at | Pages 6–7 & 11 Participant Selection (N=20,830 unweighted; weighted N=20,085); post-hoc power estimation in Statistical Analysis |
| Quantitative variables | 11 | Explain how quantitative variables were handled in the analyses. If applicable, describe which groupings were chosen and why | Pages 8–10 Birth weight categorised (Low/Normal/High); ANC visits (<8/≥8); community variables in tertiles; Table 2 |
| Statistical methods | 12 | (a) Describe all statistical methods, including those used to control for confounding | Pages 9–11 Statistical Analysis: Cox PH model stratified by country; multivariable adjustment; Stata 14.2 |
|  |  | (b) Describe any methods used to examine subgroups and interactions | Page 11 Kaplan–Meier curves stratified by maternal education, country, age, initiation, birth weight |
|  |  | (c) Explain how missing data were addressed | Page 11 Complete-case analysis; missingness described in Statistical Analysis and Table 5a footnote (religion n=1,248; birth weight n=823; ANC n=125) |
|  |  | (d) If applicable, describe analytical methods taking account of sampling strategy | Pages 9–10 svyset command; pweights; vce(cluster psu); design-adjusted chi-square tests |
|  |  | (e) Describe any sensitivity analyses | Page 11 Gompertz parametric model fitted as sensitivity analysis; AIC/BIC model comparison |
| **Results** | | | |
| Participants | 13* | (a) Report numbers of individuals at each stage of study | Page 12 & Figure 1 Participant Selection: 203,468 → 145,342 → 21,589 → 20,830 (weighted N=20,085) |
|  |  | (b) Give reasons for non-participation at each stage | Page 12 & Figure 1 Exclusions: not last-born (n=58,126); aged >5 months (n=123,753); not alive (n=759) |
|  |  | (c) Consider use of a flow diagram | Figure 1 Participant selection flow diagram provided |
| Descriptive data | 14* | (a) Give characteristics of study participants (e.g. demographic, clinical, social) and information on exposures and potential confounders | Pages 13–16 & Tables 2–3 EBF distribution by individual-level (Table 2) and community-level (Table 3) characteristics |
|  |  | (b) Indicate number of participants with missing data for each variable of interest | Page 13 & Table 5a footnote Missingness reported in Results text and Table 5a footnote: religion (n=1,248; 6.21%), birth weight (n=823; 4.10%), ANC (n=125; 0.62%) |
| Outcome data | 15* | Report numbers of outcome events or summary measures | Pages 13–14 & Table 4 Overall EBF cessation incidence 0.093/person-month; 4,732 events; 51,047 person-months; RMST 4.46 months |
| Main results | 16 | (a) Give unadjusted estimates and, if applicable, confounder-adjusted estimates and their precision (e.g. 95% confidence interval). Make clear which confounders were adjusted for and why they were included | Pages 17–20 & Tables 5a–5b Crude and adjusted HRs with 95% CIs; 18 a priori selected covariates; country-stratified Cox model |
|  |  | (b) Report category boundaries when continuous variables were categorized | Pages 8–9 & Table 2 Birth weight: <2,500 g / 2,500–3,999 g / ≥4,000 g; ANC: <8 / ≥8 visits; community variables: low/medium/high tertiles |
|  |  | (c) If relevant, consider translating estimates of relative risk into absolute risk for a meaningful time period | N/A RMST reported (4.46 person-months pooled; country range 3.78–4.95 months) as absolute summary measure |
| Other analyses | 17 | Report other analyses done — e.g. analyses of subgroups and interactions, and sensitivity analyses | Pages 20–21 & Supplementary Table S1 Gompertz parametric sensitivity analysis; country-specific shape parameters (γ); KM curves by subgroup |
| **Discussion** | | | |
| Key results | 18 | Summarise key results with reference to study objectives | Pages 22–23 Opening Discussion paragraph; five significant predictors; country heterogeneity in RMST and γ |
| Limitations | 19 | Discuss limitations of the study, taking into account sources of potential bias or imprecision. Discuss both direction and magnitude of any potential bias | Pages 31–32 Strengths and Limitations section: interval censoring; 24-hour recall; residual confounding; missing data; community-level measurement error; ANC sensitivity analysis gap |
| Interpretation | 20 | Give a cautious overall interpretation of results considering objectives, limitations, multiplicity of analyses, results from similar studies, and other relevant evidence | Pages 22–31 Full Discussion; associations framed cautiously; findings contextualised against prior SSA literature |
| Generalisability | 21 | Discuss the generalisability (external validity) of the study results | Page 32 Final sentence of Strengths and Limitations: generalisable to last-born infants 0–5 months in comparable SSA settings, 2019–2024 |
| **Other information** | | | |
| Funding | 22 | Give the source of funding and the role of the funders for the present study and, if applicable, for the original study on which the present article is based | Page 33 Declarations — Funding subsection |

**Give information separately for exposed and unexposed groups where applicable.*

*Note: An Explanation and Elaboration article discusses each checklist item and gives methodological background and published examples of transparent reporting. The STROBE checklist is best used in conjunction with this article (freely available at www.plosmedicine.org, www.annals.org, and www.epidem.com). Information on the STROBE Initiative is available at www.strobe-statement.org.*
