# Supplementary material for: Determinants and temporal trajectories of exclusive breastfeeding cessation in 16 sub-Saharan African countries: a pooled multivariable survival analysis of the demographic and health survey (2019–2024)
Source: BMC Public Health. 2026 Jul 15;26:2896. doi: 10.1186/s12889-026-28431-9 (PMC13651175; doi:10.1186/s12889-026-28431-9)
Supplement: Supplementary file 2 [file 12889_2026_28431_MOESM2_ESM.docx]

# Supplementary Material

| **Supplementary Table S1. Parametric Gompertz survival model for cessation of exclusive breastfeeding, Sub-Saharan Africa DHS 2019–2024** | | | |
| --- | --- | --- | --- |
| **Variable** | **Category** | **HR (95% CI)** | **p value** |
| Individual-level factors |  |  |  |
| Maternal age group | 15–19 years (Ref) | 1.00 (ref) | — |
|  | 20–34 years | 0.90 (0.81–1.00) | 0.054 |
|  | 35–49 years | 0.95 (0.83–1.09) | 0.435 |
| Place of residence | Urban (Ref) | 1.00 (ref) | — |
|  | Rural | 1.04 (0.95–1.13) | 0.432 |
| Maternal education level | No education (Ref) | 1.00 (ref) | — |
|  | Primary | 0.99 (0.91–1.07) | 0.724 |
|  | Secondary | 0.99 (0.90–1.08) | 0.753 |
|  | Higher | 0.82* (0.69–0.98) | 0.032 |
| Marital status | Never married (Ref) | 1.00 (ref) | — |
|  | Married/in union | 0.99 (0.90–1.10) | 0.923 |
|  | Formerly married | 0.90 (0.76–1.06) | 0.197 |
| Household wealth index | Poor (Ref) | 1.00 (ref) | — |
|  | Middle | 0.96 (0.89–1.05) | 0.375 |
|  | Rich | 0.96 (0.87–1.06) | 0.473 |
| Employment status | Not working (Ref) | 1.00 (ref) | — |
|  | Working | 0.96 (0.90–1.03) | 0.3 |
| Birth weight | Low, < 2,500 g (Ref) | 1.00 (ref) | — |
|  | Normal, 2,500–3,999 g | 1.03 (0.90–1.17) | 0.677 |
|  | High, ≥ 4,000 g | 1.22** (1.06–1.40) | 0.004 |
| Religion | Christian (Ref) | 1.00 (ref) | — |
|  | Muslim | 0.73** (0.60–0.89) | 0.001 |
|  | Other religion | 1.12 (0.81–1.54) | 0.506 |
|  | No religion | 1.13 (0.86–1.49) | 0.362 |
| Parity | One (Ref) | 1.00 (ref) | — |
|  | Two | 1.02 (0.92–1.12) | 0.757 |
|  | Three | 1.04 (0.93–1.17) | 0.472 |
|  | Four or more | 1.02 (0.92–1.13) | 0.755 |
| Early initiation of breastfeeding | After 1 hour (Ref) | 1.00 (ref) | — |
|  | Within 1 hour | 0.89** (0.84–0.95) | < 0.001 |
| Media exposure | Not exposed (Ref) | 1.00 (ref) | — |
|  | Exposed | 1.03 (0.96–1.12) | 0.411 |
| ANC visits | < 8 visits (Ref) | 1.00 (ref) | — |
|  | ≥ 8 visits | 1.16* (1.02–1.31) | 0.022 |
| Community-level factors |  |  |  |
| Community wealth level | Low (Ref) | 1.00 (ref) | — |
|  | Medium | 0.96 (0.89–1.04) | 0.355 |
|  | High | 0.99 (0.91–1.09) | 0.878 |
| Community education level | Low (Ref) | 1.00 (ref) | — |
|  | Medium | 0.98 (0.90–1.06) | 0.608 |
|  | High | 1.05 (0.96–1.15) | 0.282 |
| Community media exposure | Low (Ref) | 1.00 (ref) | — |
|  | Medium | 1.03 (0.95–1.11) | 0.48 |
|  | High | 1.00 (0.93–1.09) | 0.908 |
| Community employment level | Low (Ref) | 1.00 (ref) | — |
|  | Medium | 1.00 (0.93–1.08) | 0.989 |
|  | High | 0.95 (0.88–1.03) | 0.239 |

HR = hazard ratio; CI = confidence interval; ANC = antenatal care; Ref = reference category. Model stratified by country; clustering adjusted using robust standard errors. *p < 0.05; **p < 0.01; ***p < 0.001.
